# Supplementary material for: Body size variability across habitats in the Brachionus plicatilis cryptic species complex
Source: Sci Rep. 2022 Apr 28;12:6912. doi: 10.1038/s41598-022-10638-6 (PMC9051053; doi:10.1038/s41598-022-10638-6)

Table S1. The mean values for the limnological parameters for 25 ponds (collected at least three times in distinct periods of the year) in central and eastern Spain, inhabited by the *Brachionus plicatilis* cryptic species complex, that were initially examined in this study. Ponds in which the sediment egg banks were examined are bolded. N – the number of records per pond.

**pond**	**Temp (°C)**	**Salinity (g/L)**	**pH**	**O_2 (mg/L)**	**N**
Almenara	20.5	2.5	8.1	8.2	15
**Camino de Villafranca**	**20.7**	**48.8**	**9.2**	**10.4**	**5**
Canal Central	13.6	3.2	7.8	7.8	12
Chiprana	17.8	37.8	8.8	11.6	9
**Clot de Galvany**	**18.1**	**14.1**	**8.3**	**9.3**	**5**
**El Bassaret de L'Altet**	**19.4**	**6.7**	**7.8**	**7.5**	**7**
Estany de Cullera	19.5	18.1	8.2	6.9	3
Gallocanta	15.6	117.0	8.6	6.5	3
Grande de Villafranca	19.4	6.9	8.5	9.6	7
**Hondo Norte**	**20.3**	**10.3**	**7.8**	**4.7**	**21**
**Hondo Sur**	**20.6**	**10.8**	**7.8**	**4.4**	**24**
Horna	17.4	3.4	8.7	10.2	3
Hoya del Monte	19.6	14.2	8.6	7.4	4
Hoya Elvira	15.8	4.4	7.9	5.1	3
Hoya Rasa	15.9	36.8	9.1	5.3	3
Hoya Yerba	17.0	8.1	8.1	7.3	3
La Campana	13.9	5.4	9.3	10.7	3
Manjavacas	13.8	22.7	9.1	10.6	4
**Ontalafia**	**14.2**	**5.4**	**8.6**	**9.3**	**3**
P. Muñoz	12.2	4.4	8.8	5.1	4
**Pétrola**	**15.7**	**52.7**	**8.3**	**7.7**	**15**
**Poza Norte**	**17.8**	**10.0**	**7.6**	**5.0**	**38**
**Poza Sur**	**18.6**	**25.3**	**8.0**	**11.0**	**106**
**Salobrejo**	**14.3**	**12.9**	**8.9**	**5.3**	**12**
Taray	19.4	4.1	8.4	10.1	5

Fig. S1. An individual of the *Brachionus* sp. designated SM-X in our study. A female is carrying a resting egg internally, a phenomenon previously reported for another SM (medium-sized) species, *Brachionus ibericus*. Clone established from El Bassaret de L'Altet pond.

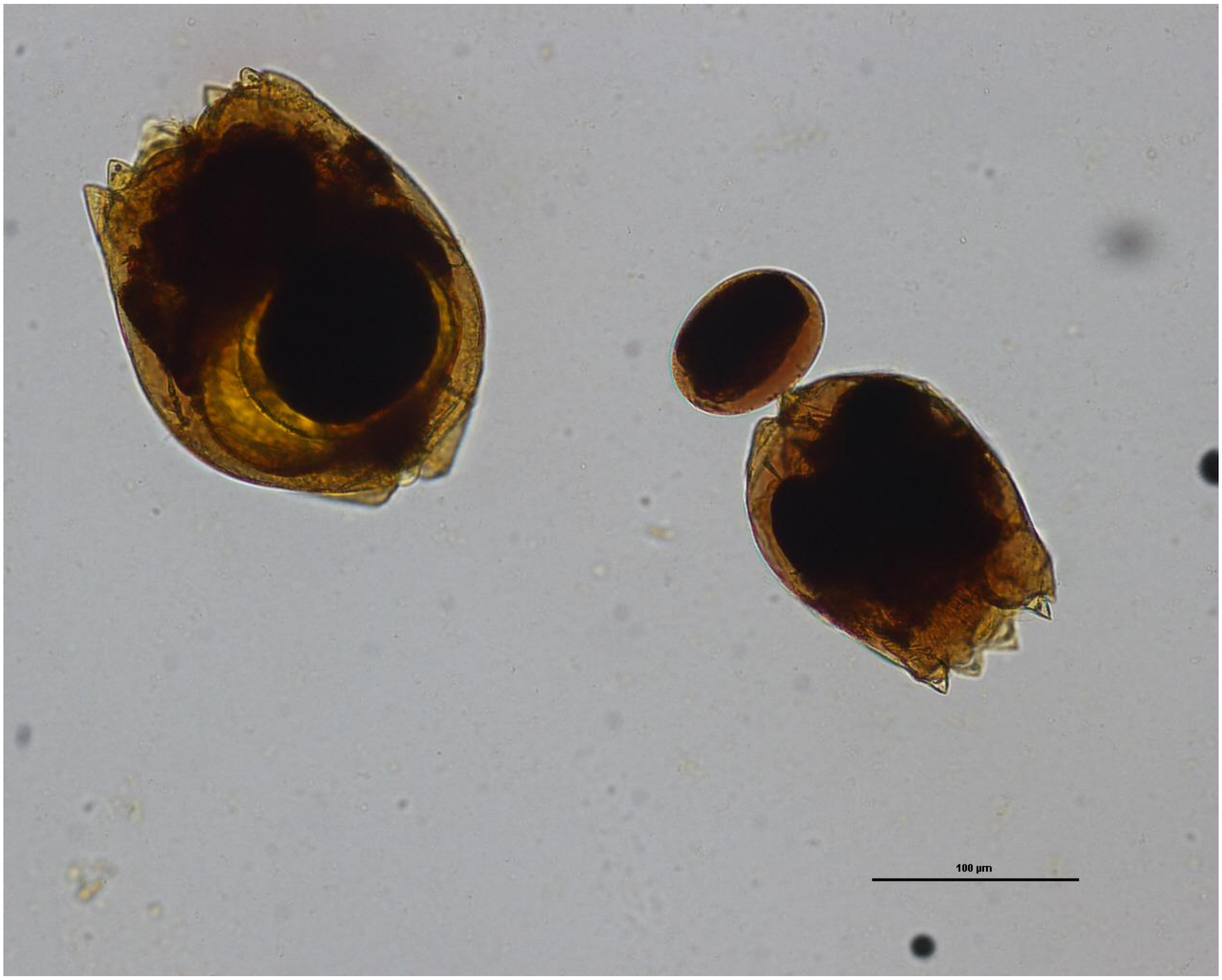

Fig. S2. Simple regressions of species body size (in ln) and four environmental factors: the oxygen concentration, salinity and temperature (driving the environmental PCA) and pH, which was of minor importance in that analysis but affected the body size of some species. The fitting line with the 0.95 Confidence Intervals shown only for significant relationships.

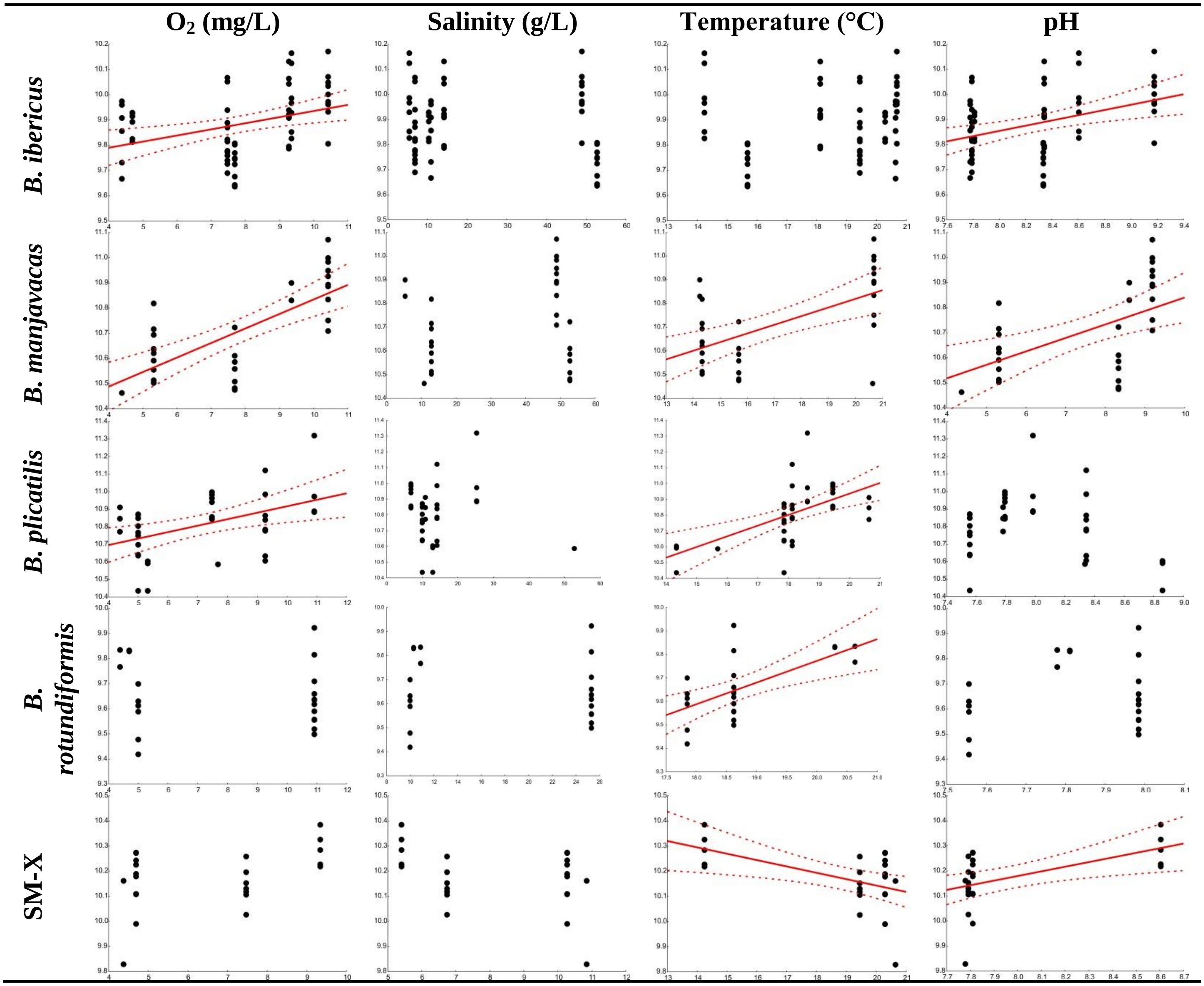

Supplement: Supplementary file 1 — Supplementary Information 1. [file 41598_2022_10638_MOESM1_ESM.pdf]
